# Impact of *Ecklonia stolonifera* extract on *in vitro* ruminal fermentation characteristics, methanogenesis, and microbial populations

**DOI:** 10.5713/ajas.19.0092

**Published:** 2019-05-28

**Authors:** Shin Ja Lee, Jin Suk Jeong, Nyeon Hak Shin, Su Kyoung Lee, Hyun Sang Kim, Jun Sik Eom, Sung Sill Lee

**Affiliations:** 1 Institute of Agriculture and Life Science and University-Centered Labs, Gyeongsang National University, Jinju 52828, Korea; 2 Division of Applied Life Science (BK21Plus) and Institute of Agriculture and Life Science (IALS), Gyeongsang National University, Jinju 52828, Korea; 3 Livestock Experiment Station, Gyeongsangnamdo Livestock Promotion Research Institute, Sancheong 52733, Korea; 4 Institute of Agriculture and Life Science, Gyeongsang National University, Jinju 52828, Korea; 5 Division of Applied Life Science (BK21Plus), Gyeongsang National University, Jinju 52828, Korea

**Keywords:** *Ecklonia stolonifera* Extract, *In vitro* Fermentation, Methane Emission, Microbial Population

## Abstract

**Objective:**

This study was conducted to evaluate the effects of *Ecklonia stolonifera* (*E. stolonifera*) extract addition on *in vitro* ruminal fermentation characteristics, methanogenesis and microbial populations.

**Methods:**

One cannulated Holstein cow (450±30 kg) consuming timothy hay and a commercial concentrate (60:40, w/w) twice daily (09:00 and 17:00) at 2% of body weight with free access to water and mineral block were used as rumen fluid donors. *In vitro* fermentation experiment, with timothy hay as substrate, was conducted for up to 72 h, with *E. stolonifera* extract added to achieve final concentration 1%, 3%, and 5% on timothy hay basis.

**Results:**

Administration of *E. stolonifera* extract to a ruminant fluid-artificial saliva mixture *in vitro* increased the total gas production. Unexpectedly, *E. stolonifera* extracts appeared to increase both methane emissions and hydrogen production, which is contrasts with previous observations with brown algae extracts used under *in vitro* fermentation conditions. Interestingly, real-time polymerase chain reaction indicated that as compared with the untreated control the ciliate-associated methanogen and *Fibrobacter succinogenes* populations decreased, whereas the *Ruminococcus flavefaciens* population increased as a result of *E. stolonifera* extract supplementation.

**Conclusion:**

*E. stolonifera* showed no detrimental effect on rumen fermentation characteristics and microbial population. Through these results *E. stolonifera* has potential as a viable feed supplement to ruminants.

## INTRODUCTION

Macro-algae are economically important and an under exploited plant resources, providing integral biomass for human foods and animal feed in recent years. Macro-algae-derived compounds have a broad range of biological activities such as antibiotic, antiviral, antioxidant, antifouling, anti-inflammatory, cytotoxic, anti-adipogenic, and antimitotic and thus confer potential health benefits [1]. In addition, macro-algae-derived compounds have been shown to increase growth rates and feed efficiency in ruminants [2]. However, they have counter-intuitively also been shown to impair fiber digestibility; thereby limiting diet digestibility [3].

Phaeophyta or brown algae are predominantly greenish brown in color due to the presence of the carotenoid fucoxanthin, and contain primary polysaccharides such as alginates, laminarins, fucans, and cellulose [4]. *Ecklonia stolonifera* (*E. stolonifera*) is a brown algae belonging to the Laminariaceae family that is commonly found in the sea forests off the coasts of Korea and Japan, growing on rocks near and below the low-tide mark on rough open coasts [5]. *E. stolonifera* has traditionally been utilized as an edible product and contains high levels of diverse phlorotannins, which are polymers of phloroglucinol found only in brown algae that have diverse biological activities, including anti-oxidative, antibacterial [5], and anti-inflammatory [6] properties. Moreover, *E. stolonifera* contains polyphenolic compounds that have been suggested to deter the grazing and growth of the seaweed’s predators [7]. However, a few studies reported that algae have potential effect on rumen fermentation characteristics and methane reduction [8,9]. Identification of feed additives that can modify the rumen microbial system to manipulate ruminal fermentation characteristics and increase the efficiency of feed utilization is an effective strategy for inhibiting ruminal methanogenesis for reducing methane emissions without an adverse effect on rumen function.

To this end, we evaluated the potential effect of *E. stolonifera* on rumen fermentation using *in vitro* gas production technique. It has previously been applied to study the fermentation kinetics of feed composition. In addition, it can allow for the rapid screening of a large number of feed additives that may have effects on gas production [10].

Therefore, this study was conducted to evaluate effects of *E. stolonifera* extracts on *in vitro* ruminal fermentation, gas profile, and changes in microbial populations. These results could help to promote *E. stolonifera* as a natural alternative for improving ruminal fermentation.

## MATERIALS AND METHODS

All experimental protocols were approved by the Animal Care and Use Committee of Gyeongsang National University (GNU-180130-A0007, Jinju, Gyeongsangnam-do, Korea).

### Ecklonia stolonifera extract preparation

*E. stolonifera* extract was obtained from the Jeju Biodiversity Research Institute (JBRI, Jeju, Korea). In brief, the plant material was washed and cut into small pieces, freeze-dried, and crushed. The plant powder was extracted with 80% methanol at room temperature (20°C) using an ultrasonic cleaner (Branson Ultrasonics Corporation, Danbury, CT, USA). After extraction, the methanol eluate solutions were filtered through Whatman No. 1 filter (Whatman International Ltd, Maidstone, UK) paper and concentrated under a vacuum.

### *In vitro* fermentation design

One cannulated Holstein cow (450±30 kg) was used as rumen fluid donors and provided with *ad libitum* access to a mineral-vitamin block and water. Twice daily (09:00 and 17:00), cows were fed 2% of their body weight in timothy hay and commercial concentrate at a 60:40 (w/w) ratio. Rumen fluid was collected before morning feedings and filtered through four layers of cheesecloth. Next, it was diluted with artificial saliva and stored at 39°C.

The chemical composition (% dry matter [DM] basis) of commercial timothy hay was as follows: moisture content, 8.87%; crude protein, 13.37%; ether extracts, 2.25%; crude fiber, 21.87%; crude ash, 8.62%; neutral detergent fiber, 53.18%; and acid detergent fiber, 30.57%.

The rumen fluid was mixed with McDougall’s buffer in a 1:2 ratio. Next, 15 mL of the mixture was dispensed anaerobically into 50-mL serum bottles containing 0.3 g of timothy for CON and *E. stolonifera* extract for treatments (TRTs) (3 mg for TRT1, 9 mg for TRT2, 15 mg for TRT3). The serum bottles were sealed anaerobically with an aluminum-capped butyl rubber stopper in pure N_2_ gas, and incubated in a shaking incubator (Jeio Tech, SI-900R, Daejeon, Korea; 120×rpm) at 39°C for 72 h. The *in vitro* fermentation experiment was a completely randomized block design and performed in triplicate, using 60 serum bottles (4 treatments × 5 incubation times × 3 replicates times).

### Determination of gas profiles and ruminal fermentation characteristics

Total gas production in the samples was measured with head space gas chromatography using a detachable pressure transducer and a digital readout voltmeter (Laurel Electronics, Inc., Costa Mesa, CA, USA). The transducer was connected to the inlet of a disposable Luer-lock three-way stopcock. Gas pressure in the headspace above the culture medium was read from the light emitting diode display unit after inserting a hypodermic syringe needle. Methane and carbon dioxide content was measured using a TCD detector with a Carboxen-1006 Plot capillary column (30 mm×0.53 mm, Supelco, Bellefonte, PA, USA), after connecting another stopcock outlet to a gas chromatograph (HP 5890, Agilent Technologies, Santa Clara, CA, USA).

Next, serum bottles were uncapped, and the culture medium was subsampled for pH (MP230, Mettler-Toledo, Columbus, OH, USA), ammonia-N and volatile fatty acid (VFA) analyses. Ammonia-N concentration was measured as optical density (OD) values at 630 nm using a UV/VIS spectrophotometer (Model 680, Bio-Rad laboratories, Hercules, CA, USA). For VFA measurements, sub-samples were centrifuged at 3,000× rpm for 3 min. The resultant supernatant was filtered using a 0.2 μm disposable syringe filter (Whatman Inc., Clifton, NJ, USA) high performance liquid chromatography (Agilent-1200, Waldbronn, Germany) using a UV/VIS detector with a MetaCarb 87H column (300 mm×7.8 mm, Varian, Palo Alto, CA, USA).

*In vitro* DM disappearance rate was determined following a modified Ørskov’s method, using nylon-bag digestion. After incubation, the nylon bag containing serum bottles was washed twice in a water-bath equipped with a Heidolph Rotamax 120 (Heidolph Instruments, Nuremberg, Germany) at 100×rpm for 30 min and then oven dried at 60°C to a constant weight. The DM disappearance was the difference in serum-bottle weight before and after incubation.

### Microbial growth rate

At the end of each fermentation period, samples were centrifuged at 3,000×rpm for 3 min to remove feed particles. The supernatant was then re-centrifuged at 14,000×rpm for 3 min to obtain a final supernatant for protein and glucose analysis. Some of the supernatant was dyed with Coomassie Blue G-250 for spectrophotometrically measuring protein content as OD at 595 nm (Model 680, Bio-Rad Laboratories, USA) [11]. For measuring glucose, 200 μL of supernatant was mixed with 600 μL of DNS solution and incubated for 5 min in a boiling water bath. Glucose concentration was the OD at 595 nm, determined with a microplate reader (Model 680, Bio-Rad Laboratories, USA) [12]. Pellets from the centrifugation were washed with sodium phosphate buffer (pH 6.5) four more times and then subjected to OD measurements at 550 nm (Model 680, Bio-Rad Laboratories, USA) to evaluate microorganism growth rates.

### Quantitative polymerase chain reaction

DNA was extracted from the incubated rumen samples using a QIAamp mini kit (QIAGEN, Valencia, CA, USA) according to the modified bead-beating protocol. Total nucleic acids were extracted by a high speed reciprocal shaker (TissueLyser; QIAGEN, USA), which retains the samples in screw-capped tubes containing ceramic and silica beads. In brief, 1 mL aliquots were taken from 15 mL of the incubated culture solution and centrifuged at 3,000 rpm for 5 min; 1 μL of the supernatant was used for nucleic acid concentration determination using a NanoDrop spectrophotometer (Thermo Scientific, Wilmington, DE, USA).

The polymerase chain reaction (PCR) primer sets were selected for amplification of general bacteria [13], ciliate-associated methanogens [14], methanogenic archaea [15], *Fibrobacter succinogenes* (*F. succinogenes*) [16], *Ruminococcus albus* (*R. albus*) [16], and *Ruminococcus flavefaciens* (*R. flavefaciens*) [16] as reported previously (Table 1).

Quantitative real-time PCR assays (CFX96 Real-Time system; Bio-Rad, USA) were conducted using the SYBR Green Supermix (QPK-201, Toyobo Co., Ltd., Tokyo, Japan) according to the methods described by Denman and McSweeney [13] and Denman et al [17]. The relative abundance of microbes was expressed according to the cycle threshold (Ct) difference as: 2^−^^Δ^^Ct (target) −^ ^Δ^^Ct (control)^. All quantitative PCR mixtures consisted of a 20 μL volume, containing forward and reverse primers, DNA template, and DNA dye SYBR Green Supermix. The PCR amplification conditions for the target DNA, including the primer annealing and extension temperatures, were the same as those reported in the corresponding reference for each primer (Table 1).

### Statistical analysis

All experimental data were analyzed using the general linear model procedure of SAS [18] as a completely randomized block design. The effects of supplementation of *E. stolonifera* extract on pH, total gas production, DM disappearance, gas profiles, VFA profiles, and methanogen diversity were compared to those of the CON group, and the data were subjected to polynomial regression to measure the linear and quadratic effects of increasing concentrations of *E. stolonifera*. Variability in the data is expressed as the standard error of the mean; p<0.05 was considered to be statistically significant, whereas p<0.10 was considered to indicate a tendency.

## RESULTS

### *In vitro* fermentation characteristics

*E. stolonifera* extract demonstrated improved cumulative gas production by mixed ruminal microorganisms as compared to that of the CON group (Table 2). However, there was no effect of *E. stolonifera* at different concentrations on pH and DM disappearance as compared with those of the CON group, except for an effect on DM disappearance at 24 h detected in the quadratic model.

As shown in Table 3, supplementation of *E. stolonifera* extract reduced the total levels of VFAs at 3 h and 48 h, acetate at 48 h, and butyrate at 3 h. Overall, supplementation of *E. stolonifera* extract decreased the acetic acid-to propionic acid ratio (A/P ratio) at 48 h as compared with that of the CON group.

Lastly, supplementation of *E. stolonifera* extract increased the methane emissions at 3 h and 12 h (linear models only); hydrogen production at 3 h, 12 h, and 72 h; and ammonia production at 72 h. By contrast, ammonia production was reduced at 24 h, respectively, as compared to those of the CON group (Table 4).

### Change in ruminal microbial diversity

*E. stolonifera* extract increased the microbial growth rate at 48 h and the glucose concentration at 3 h, while reducing the protein concentration at 12 h and at 24 h as compared with those of the CON group (Table 5).

**Figure 1:**
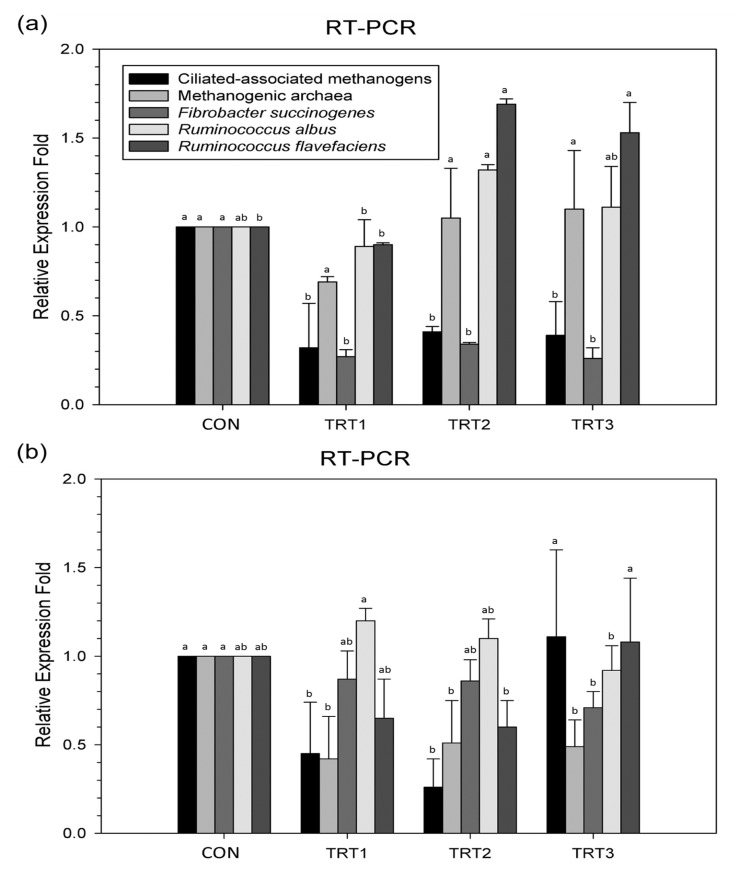
Relative quantification of rumen microorganism populations under *in vitro* ruminal fermentation for (a) 12 h and (b) 24 h. Dietary treatments were as follows: CON, basal diet (without *Ecklonia stolonifera* extract); TRT 1, 1% *Ecklonia stolonifera*; TRT 2, 3% *Ecklonia stolonifera*; TRT 3, 5% *Ecklonia stolonifera*, on a substrate (timothy hay) basis. ^a,b^ Means with different superscripts in the same row indicate significant differences (p<0.05).

The ciliate-associated methanogen and methanogenic archaea populations were reduced at 12 h (p<0.0001) and 24 h (p = 0.0164) following supplementation with various concentrations of *E. stolonifera* extract as compared with those of the CON group. In addition, *E. stolonifera* extract reduced the abundance of the major fibrolytic microorganisms such as *F. succinogenes* at 12 h (p = 0.0113) and 24 h (p = 0.0145). The proportion of *R. flavefaciens* increased at 12 h of incubation with *E. stolonifera* extract (p = 0.0001), whereas the *R. albus* population remained unchanged or slightly increased as compared with that of the CON group (Figure 1).

## DISCUSSION

Denis et al [19] reported that algae contain candidate compounds with potential to assist in ruminants feeding for improved gas production and fermentation management, within the context of dietary fiber provision. In this study, dietary fiber, as determined through the dose response of *E. stolonifera*, induced an increase in total gas production without any accompanying change in DM loss. DM disappearance only showed an effect with the addition of 1%, 3%, and 5% *E. stolonifera* extract at 24 h incubation, whereas the total gas production under all levels of *E. stolonifera* extract was higher as compared to that under incubation with Timothy hay alone at 24, 48, and 72 h, indicating the potential of this extract for improved feed efficiency [20]. The pH also remained consistent in the range of 6.49 to 7.48 for all doses of *E. stolonifera* applied during microbial fermentation, suggesting that ruminal microbial activity was not negatively affected since it was greater than the minimal pH of 5.0 to 5.5 [21].

By contrast, Wang et al [3] and Dubois et al [20] reported that brown algae species resulted in lower gas production than that of the control sample during *in vitro* ruminal fermentation. Therefore, some bioactive compounds of certain brown algae species might reduce the utilization of nutrients, thereby directly inhibiting microbial activity or indirectly by forming complexes with the nutrients [22]. Interestingly, the *E. stolonifera* extract caused a decrease in the total VFA and acetate concentrations, and resulted in a lower A/P ratio than those of the CON group at 48 h incubation, demonstrating that fermentation was affected. Secondary metabolites from *E. stolonifera* extracts have been reported to contain phlorotannins and polyphenolic compounds, which have strong antimicrobial properties and can deter the growth of the seaweed’s predators [7]. Thus, it is possible that these secondary metabolites may have induced a reduction in the total VFA concentration and altered the acetate and propionate concentrations, which are common characteristics often associated with anti-nutritional factors that interfere with ruminal fermentation [23].

With regards to emission gases, *E. stolonifera* extracts appeared to increase the *in vitro* methane emissions, and hydrogen and ammonia production, while carbon dioxide production did not increase under *in vitro* ruminal fermentation. As such, these results do not demonstrate a clear consensus trend, given that a mixed outcome was observed under different conditions. Rumen ammonia production may vary depending on the proportion of feed protein and the degradation rate; therefore, it was difficult to observe any difference in ammonia production except at 24 h and 72 h of fermentation, since timothy hay was the only substrate utilized. Wang et al [3] and Machado et al [23] reported a reduction of methane emissions when experimenting with brown algae extracts under *in vitro* fermentation conditions. Brown algae species generally show the ability to reduce methane emissions, which is most likely attributed to their phlorotannins and a range of other natural products [22,24]. However, the results from our study are in disagree with those of Wang et al [3] and Machado et al [23] as the *E. stolonifera* extracts appeared to actually increase methane emissions and hydrogen production at 3 h. This finding is in line with the results of Mitsumori and Sun [25], who suggested that ruminal methanogens utilizing mainly hydrogen would be the main source of an increase in methane emissions.

The effects of *E. stolonifera* on microbial diversity also initially appeared to be counter intuitive with the observed increase in methane and hydrogen production. *E. stolonifera* extracts reduced the populations of the ciliate-associated methanogens, methanogenic archaea, and *F. succinogenes*, while increasing the *R. flavefaciens* population as compared with those of the CON group. However, the *R. albus* population was left unchanged. Ciliate-associated methanogens may generate up to 37% of the methane produced in the rumen [26], and most methanogenic archaea can reduce CO_2_ with H_2_ to produce methane [27]. However, *F. succinogenes* is a non-H_2_-producing species [28]. Therefore, given the major reduction in the ciliate-associated methanogens and methanogenic archaea populations, a consequent reduction in methane production would be expected; however, this was not the case. *R. albus* and *R. flavefaciens* are two of the three major members of the fibrolytic microorganism population, the third being *F. succinogenes*. *R. albus* has shown great promise in the production of H_2_ from energy forage, with potential for utilizing cellulosic and hemicellulosic biomass [29]. In addition, *R. flavefaciens* normally produces succinic acid as a major fermentation product together with acetic and formic acids, H_2_, and CO_2_ [30]. As such, the increase in the *R. flavefaciens* population along with the unchanged *R. albus* population may have contributed to the observed increase in hydrogen production. Therefore, even with reductions in the ciliate-associated methanogens and methanogenic archaea populations, the increase in hydrogen availability may have allowed for increased methane emissions. Chaucheyras-Durand et al [28] showed that methane emissions clearly reduced when the dominant fibrolytic species was a non-H_2_-producing species such as *F. succinogenes*, without significantly impairing fiber degradation and fermentation in the rumen. This suggests that H_2_ is the critical factor for the microbial ecosystem in ruminants. The H_2_ produced during enteric fermentation is the precursor of methane emissions from ruminants, and thus the regulation of H_2_, rather than methane appears to be the key to controlling ruminant methane emissions.

Lastly, the *E. stolonifera* extract doses that led to higher microbial growth rates also caused higher total gas production as compared to the CON group; therefore, the rumen microorganism growth rate appears to be closely related to the total gas production and fermentation process, as suggested by Hungate [31]. In particular, the *E. stolonifera* extracts significantly increased microbial growth at 48 h as compared to that of the CON group. Moreover, our results confirmed that rumen fermentation with *E. stolonifera* extracts did not result in any negative side effects on protein or glucose concentrations throughout the experimental period. In fact, *E. stolonifera* extracts appeared to reduce the protein concentration at 12 h and 24 h. However, the protein concentration does not appear to be correlated with ammonia concentration, as Mehrez et al [32] reported that the optimal ammonia concentration could lead to maximal protein synthesis by microorganisms.

In conclusion, we demonstrated the effects of *E. stolonifera* on *in vitro* ruminant fermentation characteristics. *E. stolonifera* extracts also appear to be capable of mitigating a series of effects throughout the period of *in vitro* rumen fermentation, some of which may not be desirable. For example, *E. stolonifera* extracts could increase methane emissions and hydrogen production, which disagrees with previous observations on brown algae extracts under *in vitro* fermentation conditions. However, the changes in ruminal microbial diversity were able to partially explain the observed increase in methane and hydrogen observed with treatment of *E. stolonifera* extracts. More research is required to elucidate the potential of *E. stolonifera* for improving growth performance and methane emissions of ruminants.

## Figures and Tables

**Table 1:** Polymerase chain reaction primer sets for real-time polymerase chain reaction assays

Target species	Primer sequences (5′ to 3′)	Reference
General bacteria	F: CGG CAA CGA GCG CAA CCC R: CCA TTG TAG CAC GTG TGT AGC C	[13]
Ciliate-associated methanogens	F: AGG AAT TGG CGG GGG AGC AC R: TGT GTG CAA GGA GCA GGG AC	[14]
Methanogenic archaea	F: GGT GGT GTM GGA TTC ACA CAR TAY GCW ACA GC R: TTC ATT GCR TAG TTW GGR TAG TT	[15]
*Fibrobacter succinogenes*	F: GGT ATG GGA TGA GCT TGC R: GCC TGC CCC TGA ACT ATC	[16]
*Ruminococcus albus*	F: CCC TAA AAG CAG TCT TAG TTC G R: CCT CCT TGC GGT TAG AAC A	[16]
*Ruminococcus flavefaciens*	F: TCT GGA AAC GGA TGG TA F: TCT GGA AAC GGA TGG TA	[16]

**Table 2:** Effect of *Ecklonia stolonifera* extracts on rumen fermentation characteristics

Incubation time (h)	Treatments1)				SEM	Contrast	
	
	CON	TRT1	TRT2	TRT3		Linear	Quadratic
pH							
3	7.48	7.43	7.48	7.46	0.04	0.9938	0.6867
12	7.32	7.25	7.33	7.28	0.03	0.8845	0.8023
24	6.89	6.90	6.91	6.87	0.02	0.7286	0.2639
48	6.63	6.67	6.64	6.59	0.03	0.3154	0.2283
72	6.55	6.49	6.50	6.50	0.03	0.3132	0.3039
Gas production (mL/g DM)							
3	175.01	187.58	176.12	176.65	6.96	0.8386	0.4122
12	191.38	204.53	188.48	205.64	11.79	0.6261	0.8691
24	248.99^b^	252.53^ab^	250.31^b^	259.23^a^	2.31	0.0245	0.2764
48	282.89^b^	288.11^b^	289.38^b^	300.10^a^	3.04	0.0046	0.3933
72	288.85^b^	306.54^a^	314.04^a^	311.88^a^	3.21	0.0007	0.0148
DM disappearance (%)							
3	17.48	17.94	16.94	16.44	0.59	0.1595	0.4458
12	17.83	19.03	20.07	20.34	1.96	0.3557	0.8163
24	31.72^ab^	29.14^b^	29.99^ab^	32.52^a^	0.97	0.4734	0.0299
48	37.71	38.87	38.13	39.28	0.62	0.1911	0.9992
72	41.34	41.99	41.55	41.62	0.37	0.8107	0.4616

SEM, standard error of the mean; DM, dry matter.

1) Dietary treatments were as follows: CON, basal diet (without *Ecklonia stolonifera* extract); TRT 1, 1% *Ecklonia stolonifera*; TRT 2, 3% *Ecklonia stolonifera*; TRT 3, 5% *Ecklonia stolonifera* on a substrate (timothy hay) basis.

a,b Means with different superscripts in the same row indicate significant differences (p<0.05).

**Table 3:** Effect of *Ecklonia stolonifera* extracts on VFA by mixed rumen microbial fermentation

Incubation time (h)	Treatments1)				SEM	Contrast	
	
	CON	TRT 1	TRT 2	TRT 3		Linear	Quadratic
Total VFA concentration (mM/g)							
3	72.00^ab^	81.08^a^	67.62^b^	65.35^b^	2.85	0.0305	0.0814
12	79.12	88.59	79.13	79.05	4.04	0.6079	0.2714
24	101.92	96.37	88.34	88.23	10.06	0.3069	0.7936
48	118.93^a^	102.76^b^	95.56^b^	100.16^b^	4.44	0.0127	0.0477
72	189.75	178.98	191.12	202.28	22.52	0.6347	0.6393
Acetic acid concentration (mM/g)							
3	51.63	59.29	49.26	47.29	2.77	0.1000	0.1204
12	57.94	64.03	56.69	55.60	3.06	0.3241	0.2740
24	73.81	68.96	61.40	59.25	9.49	0.2619	0.8903
48	85.77^a^	71.11^b^	61.79^b^	67.27^b^	3.54	0.0035	0.0218
72	153.63	142.62	153.83	165.37	22.82	0.6613	0.6344
Propionic acid concentration (mM/g)							
3	11.27	13.92	11.22	10.80	2.08	0.6705	0.4808
12	11.72	15.55	13.06	13.88	1.74	0.6222	0.4129
24	18.55	18.34	16.41	19.28	0.95	0.9536	0.1441
48	22.13	21.98	21.86	21.81	1.36	0.8631	0.9670
72	24.72	25.07	24.57	25.03	0.60	0.8713	0.9336
Butyric acid concentration (mM/g)							
3	4.55^a^	3.94^b^	3.57^b^	3.63^b^	1.36	0.0016	0.0555
12	4.73	4.50	4.69	4.79	0.76	0.8562	0.7160
24	4.78	4.54	5.27	4.85	0.51	0.8139	0.9251
48	5.51	4.84	5.96	5.54	0.20	0.7575	0.8815
72	5.70	5.64	6.36	5.94	0.99	0.6516	0.7975
A/P ratio							
3	5.39	4.27	4.98	4.94	0.73	0.9177	0.6993
12	5.63	4.12	4.50	4.01	0.69	0.2236	0.5216
24	3.98	3.75	3.81	3.07	0.57	0.2803	0.6290
48	3.89^a^	3.24^ab^	2.86^b^	3.09^b^	0.60	0.0150	0.0607
72	6.24	5.74	6.26	6.62	0.96	0.7152	0.6738

VFA, volatile fatty acids; SEM, standard error of the mean; A/P ratio, acetate to propionate acid ratio.

1) Dietary treatments were as follows: CON, basal diet (without *Ecklonia stolonifera* extract); TRT 1, 1% *Ecklonia stolonifera*; TRT 2, 3% *Ecklonia stolonifera*; TRT 3, 5% *Ecklonia stolonifera* on a substrate (timothy hay) basis.

a,b Means with different superscripts in the same row indicate significant differences (p<0.05).

**Table 4:** Effect of *Ecklonia stolonifera* extracts on *in vitro* gas and ammonia production by mixed rumen fermentation

Incubation time (h)	Treatments1)				SEM	Contrast	
	
	CON	TRT 1	TRT 2	TRT 3		Linear	Quadratic
Methane emission (mL/g DM)							
3	8.79^bc^	6.63^c^	12.88^a^	11.71^ab^	1.11	0.0168	0.6698
12	10.70	13.77	15.06	17.34	2.02	0.0465	0.8510
24	22.21	15.34	27.10	20.13	4.95	0.8090	0.9923
48	27.28	24.67	26.13	22.57	2.15	0.2247	0.8331
72	31.92	32.45	26.20	30.28	7.06	0.7329	0.8072
Carbon dioxide production (mL/g DM)							
3	7.28	13.08	6.05	6.10	2.56	0.3839	0.2945
12	14.45	28.96	20.74	13.50	6.08	0.6948	0.1116
24	18.00	34.76	34.53	15.74	9.87	0.8780	0.1096
48	30.62	42.25	38.56	31.33	12.30	0.9781	0.4651
72	52.47	43.25	44.93	31.40	6.58	0.0698	0.7521
Hydrogen production (mL/g DM)							
3	1.40^b^	0.94^b^	3.04^a^	2.56^a^	0.32	0.0047	0.9803
12	3.25	3.66	4.22	4.24	0.30	0.0283	0.5330
24	4.26	4.23	5.56	4.80	0.95	0.5072	0.7113
48	5.30	4.35	7.67	6.44	2.05	0.4833	0.9470
72	8.78^b^	8.32^b^	9.48^b^	24.12^a^	3.83	0.0248	0.0838
Ammonia production (mg/dL)							
3	2.60	2.51	2.47	3.51	0.73	0.4365	0.4623
12	3.44	3.40	3.24	5.11	0.69	0.1563	0.2049
24	5.53^a^	4.91^ab^	3.51^b^	5.62^a^	0.57	0.6709	0.0447
48	9.38	9.78	9.38	9.40	0.60	0.9044	0.7609
72	13.40^b^	13.04^b^	13.82^b^	16.98^a^	0.96	0.0284	0.1061

SEM, standard error of the mean; DM, dry matter.

1) Dietary treatments were as follows: CON, basal diet (without *Ecklonia stolonifera* extract); TRT 1, 1% *Ecklonia stolonifera*; TRT 2, 3% *Ecklonia stolonifera*; TRT 3, 5% *Ecklonia stolonifera* on a substrate (timothy hay) basis.

a–c Means with different superscripts in the same row indicate significant differences (p<0.05).

**Table 5:** Effect of *Ecklonia stolonifera* extracts on rumen microbial growth rate, protein and glucose concentration

Incubation (h)	Treatments1)				SEM	Contrast	
	
	CON	TRT 1	TRT 2	TRT 3		Linear	Quadratic
Microbial growth rate (OD at 550 nm)							
3	0.33	0.33	0.34	0.25	0.03	0.1142	0.1683
12	0.35	0.31	0.36	0.30	0.02	0.4335	0.5646
24	0.28	0.32	0.28	0.28	0.03	0.8478	0.5006
48	0.32^b^	0.38^ab^	0.37^ab^	0.43^a^	0.03	0.0295	1.0000
72	0.27	0.29	0.33	0.24	0.03	0.7287	0.0977
Protein concentration (mM/g)							
3	0.14	0.16	0.15	0.15	0.01	0.5664	0.5868
12	0.19^a^	0.16^b^	0.15^b^	0.16^b^	0.01	0.0323	0.0490
24	0.24^a^	0.19^b^	0.18^b^	0.19^b^	0.01	0.0017	0.0089
48	0.27	0.20	0.18	0.21	0.03	0.2304	0.1758
72	0.28	0.23	0.23	0.23	0.04	0.4818	0.6027
Glucose concentration (mL/mg)							
3	0.10^ab^	0.10^b^	0.11^ab^	0.12^a^	0.01	0.0418	0.0868
12	0.11	0.10	0.12	0.13	0.01	0.1268	0.3292
24	0.14	0.12	0.16	0.15	0.03	0.5625	0.8210
48	0.17	0.15	0.16	0.16	0.03	0.9801	0.7620
72	0.34	0.19	0.31	0.27	0.13	0.8968	0.6775

SEM, standard error of the mean; OD, optical density.

1) Dietary treatments were as follows: CON, basal diet (without *Ecklonia stolonifera* extract); TRT 1, 1% *Ecklonia stolonifera*; TRT 2, 3% *Ecklonia stolonifera*; TRT 3, 5% *Ecklonia stolonifera* on a substrate (timothy hay) basis.

a,b Means with different superscripts in the same row indicate significant differences (p<0.05).
